# Therapeutic Effect of Pericytes for Diabetic Wound Healing

**DOI:** 10.3389/fcvm.2022.868600

**Published:** 2022-05-13

**Authors:** Kyeong Mi Kim, Hyun-Ju An, Sang-Hoon Kim, JuHee Kim, Changgon Sim, Jaemin Lee, Sin Hyung Park, Hyun Il Lee, Inseok Jang, Soonchul Lee

**Affiliations:** ^1^Department of Laboratory Medicine, CHA Ilsan Medical Center, CHA University School of Medicine, Goyang-si, South Korea; ^2^Department of Orthopaedic Surgery, CHA Bundang Medical Center, CHA University School of Medicine, Seongnam-si, South Korea; ^3^Department of Cardiology, CHA Bundang Medical Center, CHA University School of Medicine, Gyeonggi-do, South Korea; ^4^CHA Graduate School of Medicine, Gyeonggi-do, South Korea; ^5^Department of Orthopaedic Surgery, Bucheon Hospital, Soonchunhyang University School of Medicine, Gyeonggi-do, South Korea; ^6^Department of Orthopedic Surgery, Ilsan Paik Hospital, Inje University, Gyeonggi-do, South Korea

**Keywords:** ischemic foot ulcer, pericyte, angiogenesis, diabetes mellitus, wound healing

## Abstract

**Objective:**

Numerous attempts have been made to devise treatments for ischemic foot ulcer (IFU), which is one of the most severe and fatal consequences of diabetes mellitus (DM). Pericytes, which are perivascular multipotent cells, are of interest as a treatment option for IFU because they play a critical role in forming and repairing various tissues. In this study, we want to clarify the angiogenic potential of pericytes in DM-induced wounds.

**Methods:**

We evaluated pericyte stimulation capability for tube formation, angiogenesis, and wound healing (cell migration) in human umbilical vein endothelial cells (HUVECs) with *in-vivo* and *in-vitro* models of high glucose conditions.

**Results:**

When HUVECs were co-cultured with pericytes, their tube-forming capacity and cell migration were enhanced. Our diabetic mouse model showed that pericytes promote wound healing *via* increased vascularization.

**Conclusion:**

The findings of this study indicate that pericytes may enhance wound healing in high glucose conditions, consequently making pericyte transplantation suitable for treating IFUs.

## Introduction

Diabetes mellitus (DM) is a highly prevalent metabolic disorder that affected approximately 9.3% of the global adult population (20–79 years) in 2019 according to the International Diabetes Federation Diabetes Atlas (1). DM prevalence has risen dramatically due to global changes in nutrition and lifestyle, was estimated to affect 425 million adults in 2017, and is expected to affect 629 million patients by 2045 (2).

Ischemic foot ulcer (IFU) is a common, clinically important complication of DM, with approximately 10–25% of diabetes patients developing an IFU during their lifetime (3–6). IFU is a significant cause of morbidity and hospitalization for diabetic patients (7). If necessary care is not provided, it can lead to infection, gangrene, amputation, and death. It is estimated that approximately 50–70% of all lower-limb amputations are due to IFU (8). Along with pain, infection, amputation, and decreased mobility, IFU also has significant economic, social, and psychological consequences (9). Current treatment guidelines of IFU recommend debridement, infection control, revascularization, and offloading pressure (10). However, ischemia, infection, neuropathy, and metabolic problems delay wound healing, posing a significant obstacle for patients and clinicians (11). Various methods have been attempted to overcome IFU, which is challenging to treat. Particularly, several ways have been proposed to promote and maintain angiogenesis using stem cells, nanoparticles, and scaffolds. However, these may be inadequate in attracting sufficient host vasculature to promote tissue regeneration in a clinically meaningful manner (12–15).

Recent improvements in our knowledge of the cellular and molecular complexity of wound healing have revealed coagulation, inflammation, cell migration, and proliferation as essential processes in the remodeling and repair of tissue (16). Pericytes, which are perivascular multipotent cells, play a critical role in forming and repairing diverse tissues by cell migration, regeneration, and contributing to cell signaling (17). Pericytes have been previously documented to behave as mesenchymal stem cells (18). Most human dermal pericytes have been reported to differentiate into bone, fat, and cartilage lineages (19). Additionally, pericytes contribute to regeneration observed in myogenesis, adipogenesis, neurogenesis, tissue fibrosis by proliferation, and diverse molecular signaling with other cells (18, 20–22). Notably, pericytes show angiogenic potential by interacting with endothelial cells through several molecules such as vascular endothelial growth factor, transforming growth factor-beta, platelet-derived growth factor beta, and angiopoietins, as well as signaling pathways involving Notch and ephrins (23). These recent findings suggest distinct roles for pericyte subsets, enabling the development of novel treatment methods. However, to the best of our knowledge, no studies have been conducted considering pericytes as an IFU treatment. This study aims to investigate the therapeutic effects of pericytes on IFU *in vitro* and *in vivo*.

## Materials and Methods

### Cell Culture

Human umbilical vein endothelial cells (HUVECs) and human pericytes underwent VascuNet™ Pericyte Co-Culture Assays (ESI BIO, USA) and were grown in VascuNet Basal Assay Medium supplemented with recombinant human vascular endothelial growth factor (VEGF), epidermal growth factor (EGF), insulin-like growth factor-1 (IGF-1), fibroblast growth factor (FGF) basic, ascorbic acid, heparan sulfate, hydrocortisone hemisuccinate, L-glutamine, 5% fetal bovine serum (FBS), penicillin (100 U/ml), and streptomycin (10 μg/ml) at 37°C in a humidified incubator containing 5% CO_2_.

### Isolation of Mouse White Adipose Tissue Adipocytes

Subcutaneous white adipose tissue (scWAT) and visceral gonadal WAT (gWAT) white fat pads were dissected and processed for cell isolation. Mouse adipocytes were obtained according to a previously reported method with minor modifications (24). Mouse WAT fat pads were dissected and gently minced with scissors for 2–5 min or until no visible tissue fragments remained. The minced tissue was digested in a shaking water bath at 37°C and 170 rpm with 10 ml of phosphate-buffered saline (PBS) containing 1% bovine serum albumin (BSA), 400 nM of adenosine, 50 μg/ml of gentamicin, 2.5 mg/ml of dispase II, and 10 mM of CaCl_2_ in the presence of collagenase D (Roche). Minced WAT was digested using collagenase, then the adipocytes were washed three times with 40 ml wash buffer [1% bovine serum albumin (BSA), 400 nM adenosine, 50 μg/ml gentamicin in PBS, pH 7.4] at room temperature. The infranatant was removed using a syringe and needle after allowing the cells to float for more than 5 min after each round of washing. After removing the wash buffer from the last round, isolated adipocytes were filtered using a 100- or 300-μm nylon mesh strainer and placed in Dulbecco's PBS (DPBS) for staining. Primary adipocytes were not centrifuged, since this would result in greater cell breakage and sample detritus. When fixation was required, adipocytes were fixed with fixative buffer (2% paraformaldehyde and 1% sucrose in DPBS) for 30 min with gentle agitation. The cells were resuspended in the medium after washing with wash buffer (24).

### Flow Cytometry Analysis

We applied three markers to flow cytometry (FCM): CD146-fluorescein isothiocyanate (FITC), nestin-phycoerythrin (PE), and NG2-PE to assess and sort the pericyte component of cultured mouse WAT adipocytes. Cells were collected from culture dishes using trypsin ethylenediaminetetraacetic acid (EDTA), fixed in 4% paraformaldehyde at pH 7.4, and dissolved in PBS. For FCM, a single cell was treated with the appropriate primary antibodies for 30 min and then rinsed with PBS supplemented with 1% FBS. After washing twice to remove the primary antibody, samples were re-incubated with an FITC- or PE-conjugated secondary antibody (Invitrogen, USA) at a dilution of 1:500 for 20 min. Prior to initiating FCM, cells were washed twice more with wash buffer. FCM was performed using FACSCalibur (Beckman Coulter, California, USA) (25).

### Vascular Tube Formation

A Matrigel matrix (Corning, USA) was thawed on a 24-well plate. After 1 h at ambient temperature, the coated 24-well plate was placed in an incubator at 37°C with a humidified environment of 5% CO_2_ for 1–2 h prior to plating. Each well contained 300 μl of the human umbilical vein endothelial cells (HUVECs) and pericytes (10:1 ratio) cell suspension in the VascuNet Basal Assay Medium (ESI BIO, USA) (26).

### Wound Healing Assay

Cell migration was determined using a wound healing test. Clear lines were formed using a sterile 200 μl pipette tip after cells were planted at the same density per well for 12 and 24 h. The cells were then grown continuously for 12–24 h in media supplemented with 5% FBS. At 0, 12, and 24 h following initiation of the wound, photomicrographs with the scratches were taken at prefixed positions. ImageJ was used to determine the migration distance. Each experiment was conducted a minimum of three times. In each scenario, the mean was calculated as the average of the experimental repetitions (27).

### Diabetes Induction and Animal Monitoring

For 5 days, mice were intraperitoneally injected with a single high dose of 50 mg/kg of streptozotocin (STZ). Shock fatalities were reduced by substituting 5% sucrose water for drinking water. The tail was pricked with a lancet to draw blood. After 2 weeks of STZ, body weight and blood glucose levels were examined daily until a diabetic condition was confirmed. Diabetic mice were defined as those with a glucose level of more than 300 mg/dl. Mice that had a successful course following diabetes induction were then assigned to investigations.

### Murine Diabetic Wound Healing Model

All mice were kept in compliance with the National Institutes of Health (NIH) and institution-approved animal care protocols at the CHA University Veterinary Service Center. The CHA Administrative Panel on Laboratory Animal Care authorized all operations. Male mice aged 8–10 weeks (Orient Bio, Korea) were divided into four groups: “untreated control” (control), “pericyte injection” (pericyte), “diabetes mice without pericyte injection” (DM without pericyte), and “diabetic mice with pericyte injection” (DM with pericyte) (*n* = 10 wounds per treatment group). Under anesthesia, the dorsal surface of the mouse was shaved. On either side of the midline, two full-thickness incisions 4 mm in diameter were produced. To avoid wound contraction, the wounds were circumscribed with donut-shaped silicone splints. A 10-μl mixture was pipetted over a type I collagen sponge and incubated for 1 day to completely remove the sponge (Advanced BioMatrix, California, USA). All the wounds were then treated with a commercially available clear film covering 3M Tegaderm Film (Minnesota, USA). Photographs were taken on each day and the wound area was measured using ImageJ software (NIH, Maryland, USA). At different time points, the percentage of the original wound area was calculated with reference to the wound area on the day of surgery (28).

### Histological Procedures

A total of 14 days after wounding, wound beds and underlying muscle were removed, fixed for 4 h in 10% formalin (Sigma-Aldrich), treated with 100% ethanol and xylenes, and embedded in paraffin. Serial sectioning of 10 wounds (7 μm) perpendicular to the wound surface was performed in the rostral-to-caudal direction for each group. For wound analysis, every tenth segment of the whole wound bed was stained with H&E and Masson's trichrome. Immunohistochemical analysis determining the blood vessel formation capacity of the pericytes was conducted according to the manufacturer's protocol using a Ready-to-use IHC/ICC kit (BioVision Incorporation). Tissues were preserved for 24 h at room temperature with 4% formaldehyde. Tissues fixed in paraffin were sectioned into 5 μm thick slices, deparaffinized, rehydrated, and subjected to antigen retrieval. After blocking endogenous peroxidase activity with 3% hydrogen peroxide (H_2_O_2_), the slides were treated at room temperature with anti-Ki67 (Abcam, ab15580) and anti-CD31 (Abcam, ab28364) antibodies. Additionally, angiogenesis was identified by using immunohistochemistry with antibodies to α-smooth muscle actin (α-SMA), collagen 1 (Col 1), and Nestin. Following incubation with horseradish peroxidase (HRP)-anti-mouse immunoglobulin G (IgG) polymer and washing with PBS, the tissue sections were treated at room temperature with 3,3′-diaminobenzidine. The images were taken with an Eclipse Ts2 microscope at 100 × magnification.

### Statistical Analyses

GraphPad Prism program version 6 (GraphPad Software Incorporation, San Diego, California, USA) and R software were used to conduct all the statistical analyses (version 3.2.4; R Foundation for Statistical Computing). Additionally, when only two groups were assessed, the Mann–Whitney *U* test was utilized to determine significance. The significance level was set at *p* ≤ 0.05 (^**^p ≤ 0.01, ^****^p ≤ 0.0001). Unless otherwise specified, all *in-vitro* studies were repeated at least three times.

## Results

### Diabetes Mellitus Impairs Pericyte Markers

**Figure 1:**
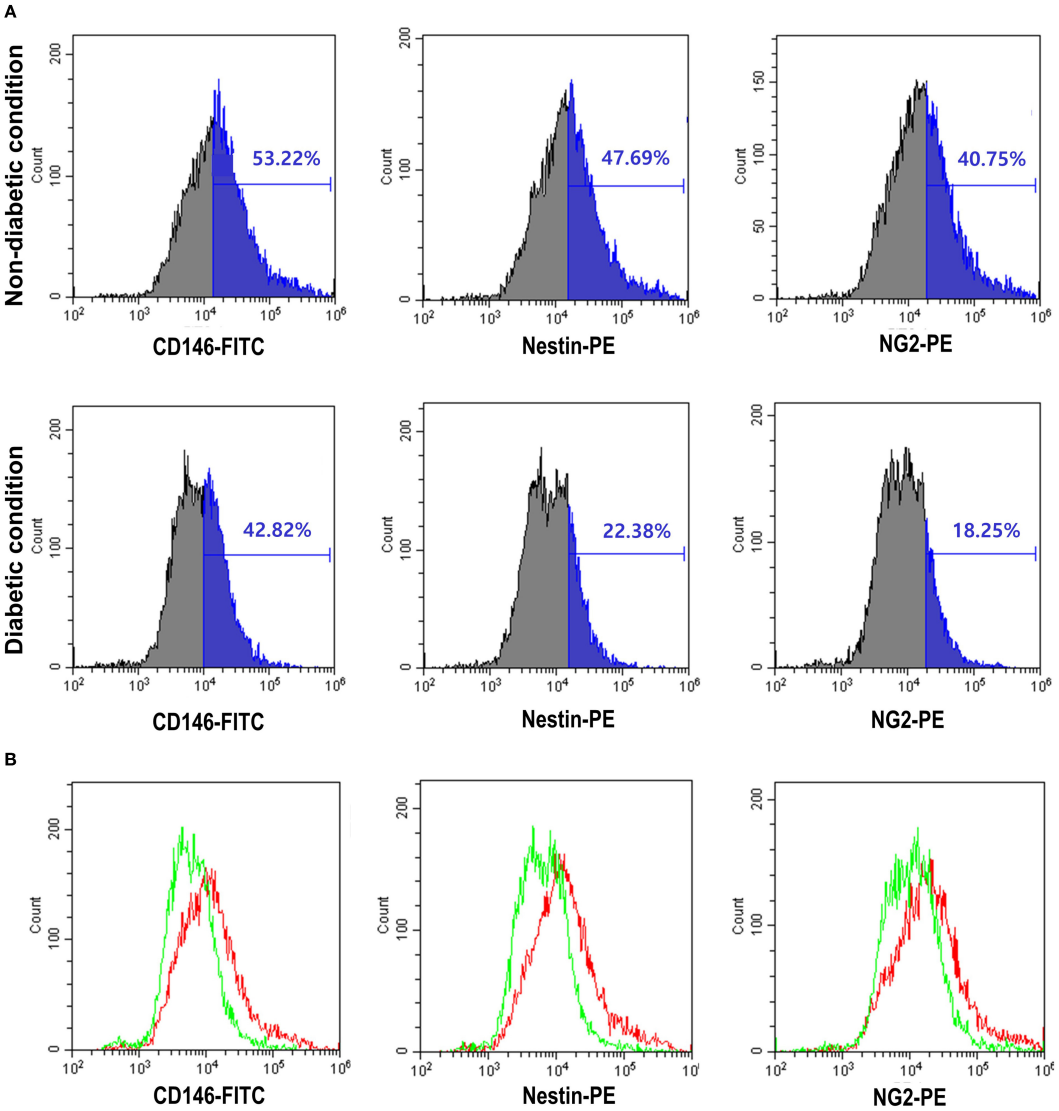
The expression of pericyte markers by flow cytometry was reduced in the diabetic condition. **(A)** Analysis of pericyte surface markers in the non-diabetic and diabetic conditions using flow cytometry [CD146-fluorescein isothiocyanate (FITC), Nestin-phycoerythrin (PE), and NG2-PE]. The results provided here are representative of those obtained in three independent studies. In the diabetic condition, Nestin and NG2-PE were seen to decrease by more than half. **(B)** Overlay of diabetic condition (green line) and non-diabetic (red line) condition flow cytometry. The diabetic condition showed the three pericyte markers to be decreased.

We used three-marker polychromatic FCM to assess and sort the pericyte component of cultured mouse WAT adipocytes. Figure 1 shows the expression degree of the three pericyte markers. The frequencies of pericyte markers in the non-diabetic and diabetic conditions were as follows: CD146-FITC, 53.22 and 42.82%; Nestin-PE, 47.69 and 22.38%; and NG2-PE, 40.75 and 18.25%, respectively (Figure 1A). FCM results show decreased levels of the three indicators in diabetic mice compared to those in nondiabetic mice. The difference in expression levels of these three markers is shown in the overlay histogram (Figure 1B).

### Pericytes Increase the Tube Formation of Human Umbilical Vein Endothelial Cells Cultured in a High Glucose Condition

**Figure 2:**
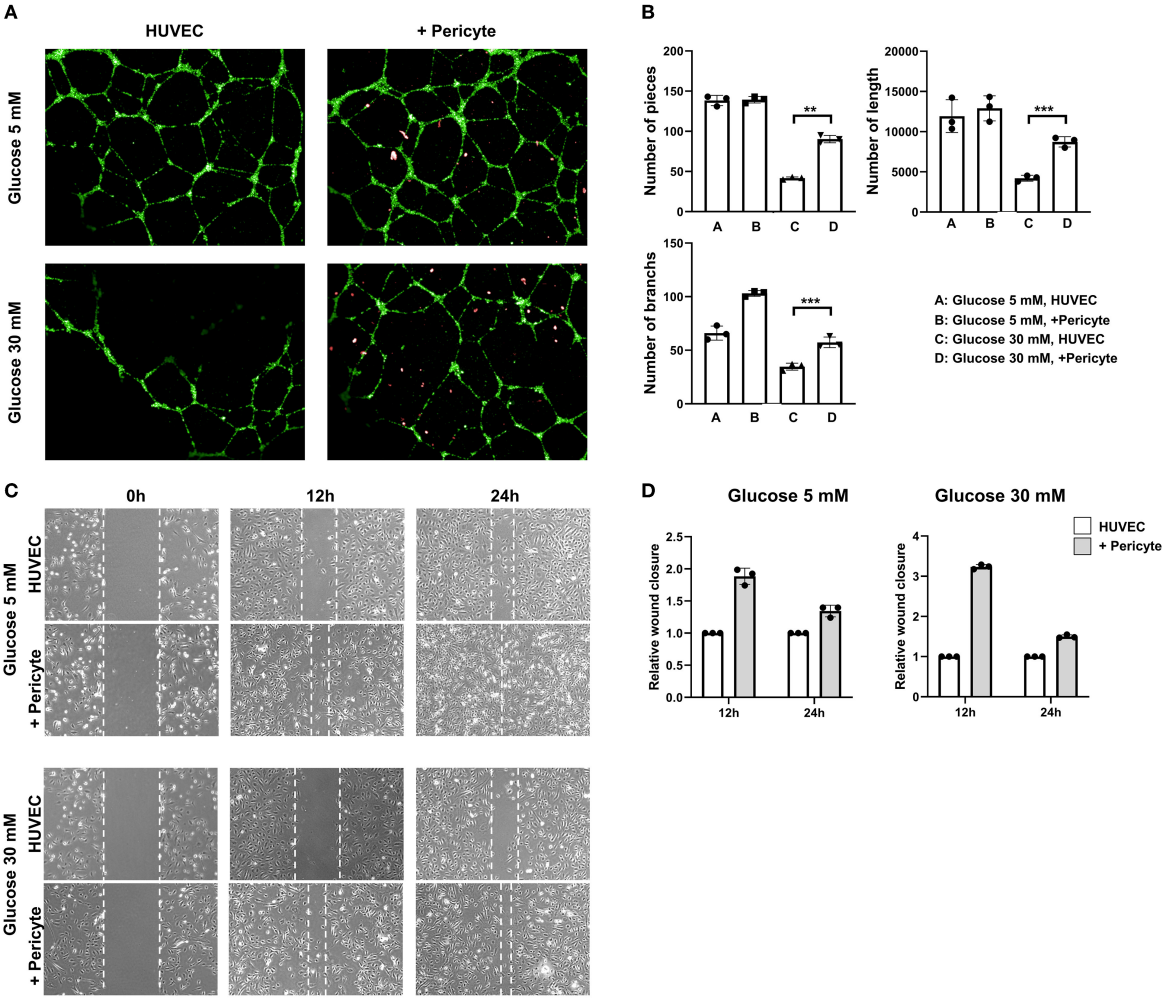
The group with pericytes showed improved tube formation and cell migration ability in the high glucose condition. **(A)** The *in-vitro* vascular, tube formation assay showed effects of exposure on FITC-tagged human umbilical vein endothelial cell (HUVEC) tube formation with and without PE-tagged human pericyte cells. The optimal observation time for tube formation was 8 h after seeding. In high glucose conditions, HUVECs with pericytes (10:1 ratio) showed active tube formation even compared to the without pericyte group. **(B)** The number of pieces in the assay was measured. (**P < 0.01 and ***P < 0.001 according to the Student's *t*-test). HUVEC tube formation capacity showed no significant difference in low glucose conditions. However, in high glucose conditions, the group with pericytes had a statistically significant increase in tube formation. **(C)** Photomicrographs were taken at 0, 12, and 24 h following initiation of the wound. HUVEC migration, often termed as wound closure ability, was more remarkable in the group with pericytes in both the high and low glucose conditions. **(D)** The quantitative approach applied to the wound closure assay, showing a histogram calculated based on the group breath ratio of the control group and the group with pericytes.

At 5 mM glucose concentrations, the capillary formation capacities of the HUVEC group and the HUVEC-with-pericytes group were similar, as were numerical data such as number of pieces, number of branches, and total length. However, the results for 30 mM glucose concentration demonstrated a substantial change, involving thinning of the capillaries and a decrease in the number of branches compared to the observations for the 5 mM glucose concentration (Figure 2A). At a 30 mM glucose concentration, the number of pieces and branches increased significantly in the HUVEC-with-pericytes group compared to that in the HUVEC group (Figure 2B).

### Pericytes Promote Cell Migration

We evaluated the contribution of morphological differences induced by pericytes to cell migration using a wound healing assay and time-lapse microscopy. At 5 mM glucose concentrations, the HUVEC-with-pericytes group showed faster wound healing, almost completing the closure by 24 h, whereas the HUVEC group progressed slowly. However, at 30 mM glucose conditions, slow, almost negligible migrations were seen in the HUVEC group, which showed significant progress with the addition of pericytes (Figure 2C). Analysis of wound closure was performed by a semi-quantitative approach, using the breadth of the wounds to estimate the relative wound closure. At a 30 mM glucose concentration, pericyte-treated cells migrated at a remarkably faster rate than that of the HUVECs only in the initial time period (Figure 2D).

### Pericytes Accelerate the Healing of Cutaneous Wounds in an *in-vivo* Diabetes Mellitus Mouse Model

**Figure 3:**
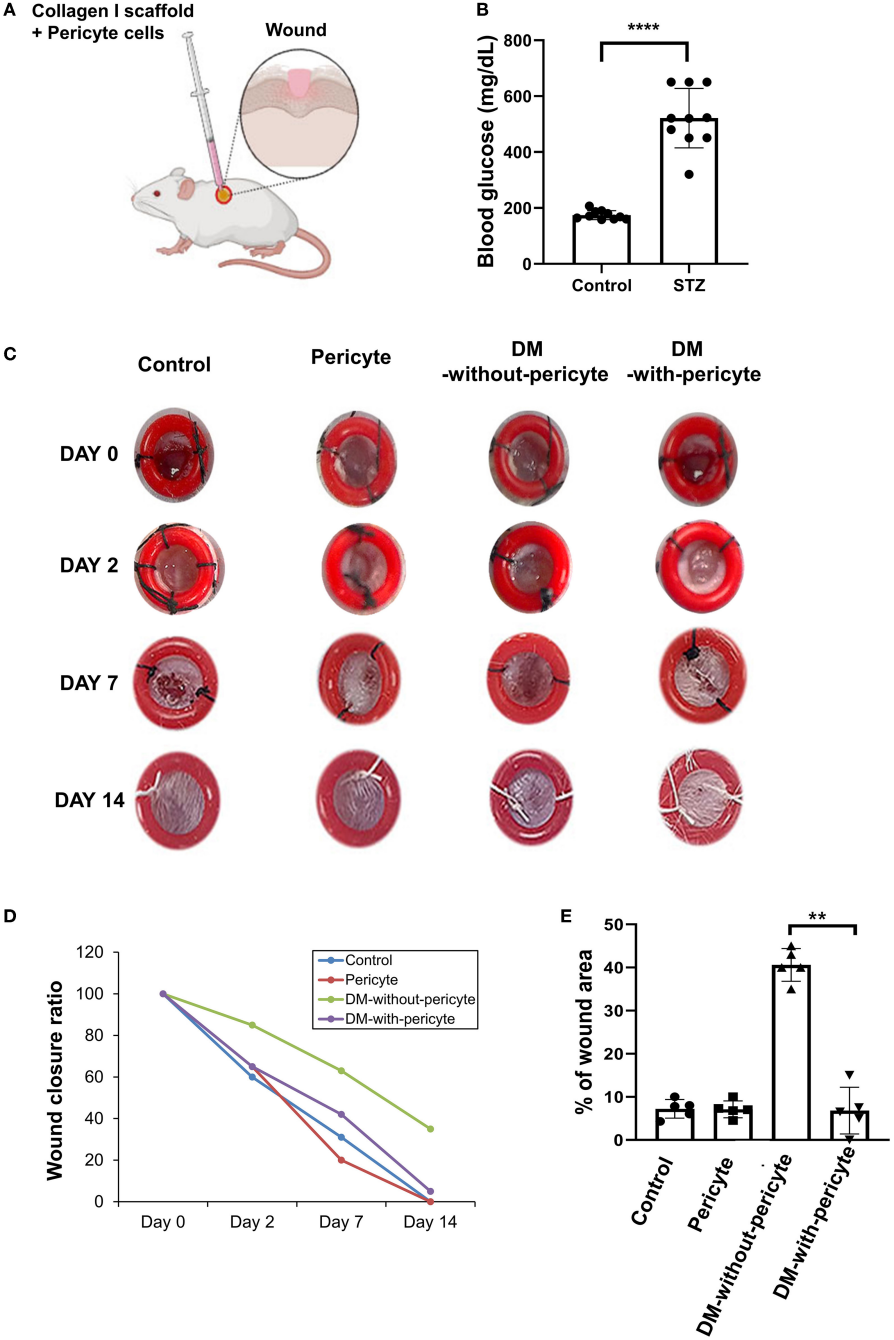
The diabetes mellitus (DM)-with-pericyte group exhibited substantial wound healing in a mouse wound model. **(A)** Illustration of injection of collagen type 1 scaffold + 5 × 10^5^ pericytes in the mouse wound. **(B)** Streptozotocin (STZ)-induced diabetic mice on blood glucose confirmed successful DM induction (****P < 0.0001). DM-induced mice (*n* = 10), control (*n* = 10). **(C)** Incisions with a diameter of 4 mm on either side of the midline were produced. To avoid wound contraction, the wounds were circumscribed with donut-shaped silicone splints. As observed on day 7, wounds of the DM-with-pericyte group healed, whereas those of the DM-without-pericyte and control groups did not heal. **(D)** The Y-axis of the graph represents the wound size, when measured on days 0, 2, 7, and 14. It was verified that all the groups, except the DM-without-pericyte group, had wound closure by the time of sacrifice on day 14 and approximately 3.6 mm of the wound of the DM-without-pericyte group remained. The pericyte and DM-with-pericyte groups showed the same wound size on days 0, 2, 7, and 14. **(E)** Quantification of wound size at sacrifice time points (**P < 0.01). In comparison to the DM-without-pericyte group, the DM-with-pericyte group exhibited substantial wound healing.

The effects of the pericyte treatments were assessed *in vivo* using a diabetic mouse model. The development of diabetes by STZ treatment was verified by blood glucose measurements (Figures 3A,B). Figure 3C shows the excisional wounds on 0, 2, 7, and 14 days after operation in the four groups. On day 2, it was confirmed that the wound size decreased in the DM-with-pericyte group compared to the DM-without-pericyte group. On day 14, the wound area of the DM-with-pericyte group was 0% and that of the DM-without-pericyte group was 36%, showing a statistically significant difference (Figures 3D,E).

**Figure 4:**
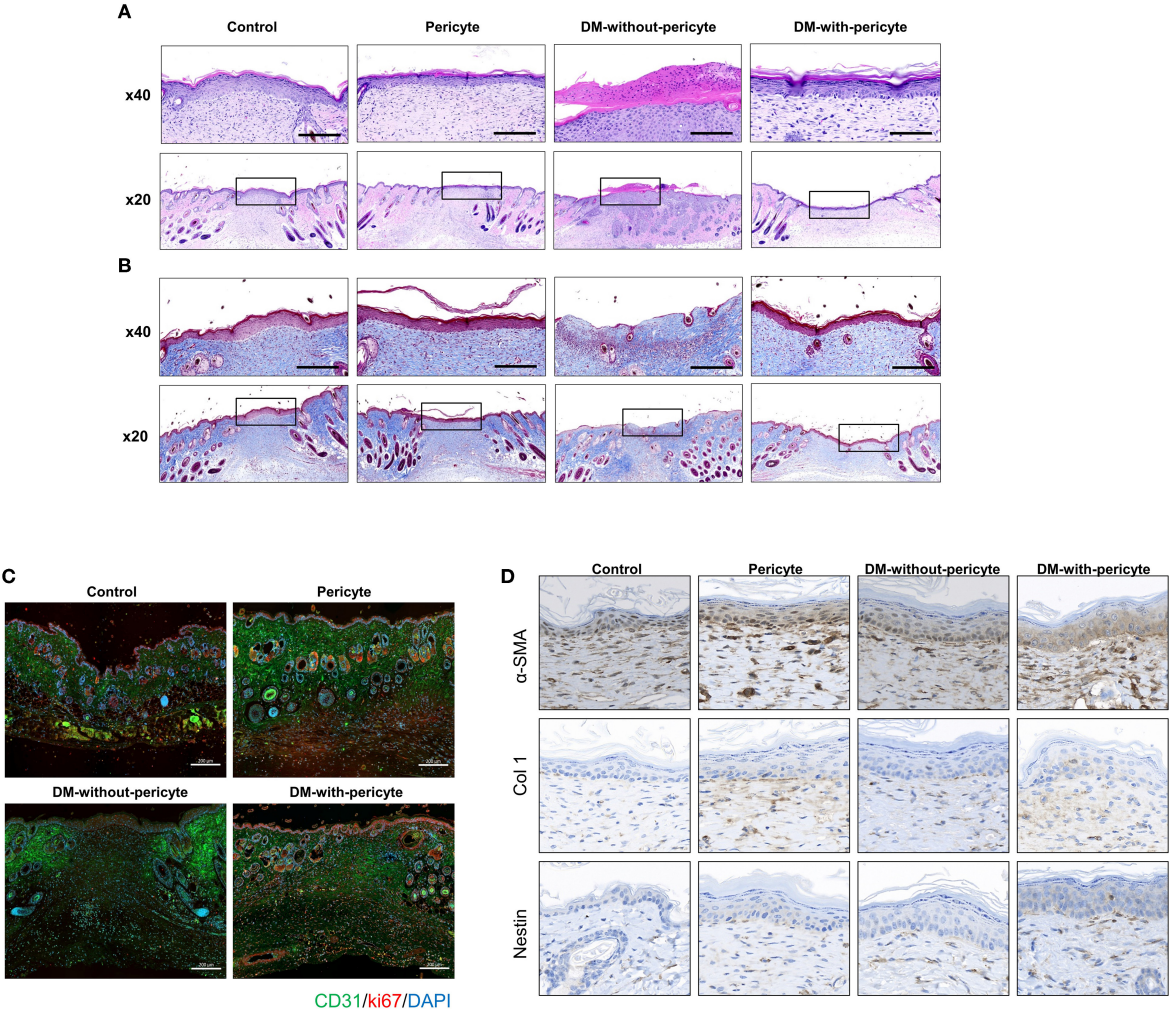
The DM-with-pericyte group showed successful repair of the collagen layer and higher blood vessel formation capacity compared to the DM-without-pericyte group. **(A)** H&E staining. **(B)** Masson's trichrome staining for wound analysis at 14 days. The black square indicates the regenerated dermal collagen layer. Scale bars = 200 μm. **(C)** CD31 and Ki67 immunofluorescence of wounds at 14 days. In diabetes wounds, new vessels were significantly less visible than in normal mice. However, when diabetic mice were injected with pericytes, new vessels were observed more often. **(D)** α-smooth muscle actin (α-SMA), collagen 1 (Col 1), and Nestin immunohistochemistry of wounds at 14 day. Scale bar = 50 μm.

Blood vessels create continuous networks parallel to the skin's surface, which can be seen by techniques used to assess blood vessel health, such as H&E and Masson's trichrome staining of dermal scars. The DM-without-pericyte group showed incomplete blood vessel formation and no regeneration of the dermal collagen layer. However, the other three groups—control, pericyte, and DM-with-pericyte—were able to repair their collagen layers successfully (Figures 4A,B). CD31 and Ki67 co-staining were used to assess angiogenesis. Both the markers were remarkably increased in the DM-with-pericyte group compared to those in the DM-without-pericyte group (Figure 4C). In addition, immunohistochemical analysis showed that α-SMA, Col 1, and Nestin expression were higher in the DM-with-pericyte group compared to those in the DM-without-pericyte group, indicating that angiogenesis was induced in these groups (Figure 4D).

## Discussion

Numerous attempts have been made to devise treatment for IFU, a severe and fatal consequence of DM, including cell therapy and treatment with various pharmaceuticals. However, insufficient angiogenic capacity limits their usage in practice, necessitating further study and development of novel therapies. Angiogenic potential of pericytes is drawing interest in this context, which we want to elucidate in this study with regard to its therapeutic application for DM-induced wounds. Herein, we evaluated pericyte stimulation capability for tube formation, angiogenesis, and wound healing (cell migration) in HUVECs, an endothelial progenitor cell (EPC) line, with models of high glucose conditions *in vivo* and *in vitro*.

We discovered that high glucose substantially decreased tube development, angiogenesis, and wound closure. Our findings corroborate prior research suggesting that hyperglycemia causes premature senescence of EPCs and raises levels of reactive oxygen species and inflammation (29–33). However, recent studies of muscular, neural, and vascular regeneration capacity of pericytes suggest that pericytes could reverse this problem. Dellayalle et al. found that pericytes create satellite cells that are actually muscle stem cells and pericytes are promising candidates for future cell-based therapies to treat muscular dystrophies (34). When grown under optimal conditions, type 2 pericytes generate neural progenitors that resemble brain NG2-glia (35). Pericytes have neural stem cell-like properties that allow them to be reprogrammed to differentiate into other neurovascular cells, thereby making them a treatment option for neurovascular diseases (36). Additionally, several studies report pericytes to have a role in wound healing by contributing to angiogenesis (37, 38). Birbrair et al. demonstrated that type 2 pericytes increase ligated femoral artery blood flow in ischemic mice legs. These findings suggest type 2 pericytes to be helpful for vascular treatment in ischemic diseases (39). Kang et al. demonstrated tumor necrosis factor (TNF) to have antiangiogenic characteristics at high doses and proangiogenic capabilities at low concentrations and pericytes that possibly regulate the TNF concentration to be proangiogenic (40). Recently, various applications of pericytes have been proposed. Co-culturing of mouse primary brain endothelial cells and brain pericytes on a three-dimensional collagen scaffold by building an organ-specific microfluidic platform that replicates the angiogenic milieu *in vivo* has been reported (41). The substantial angiogenic ability of pericytes is also attracting attention as a method to suppress tumor growth (42).

This study shows pericytes to have the potential to facilitate HUVEC regeneration despite DM conditions. The purpose of this study was to determine the therapeutic benefits of pericytes for stress-induced senescence of HUVECs produced by high glucose. The tube-forming capacity of HUVECs was enhanced on being co-cultured with pericytes. Additionally, the wound healing assay indicated the presence of pericytes to alleviate high glucose-induced stress in HUVECs, thus demonstrating their angiogenic potential. The findings of this study are consistent with those of other studies on the healing potential of pericytes.

However, this study has a limitation of not having results regarding pericyte cytokines or cell signaling mechanisms. This will be addressed in subsequent studies. Moreover, to apply findings of animal studies on pericyte subtypes to humans, it is necessary to verify particular markers for pericyte subpopulations in human tissues by microarray research to decipher their endogenous physiological response to physiological and pathological circumstances. According to previous articles, there is a decrease in pericytes in diabetic patients and endogenous pericytes help in wound healing and vascularization (43, 44). However, this study is meaningful in that it identifies heterogeneous and distinct roles of pericytes and their potential therapeutic application for IFU treatment.

In conclusion, this study shows pericyte therapy to be a successful therapeutic option for patients with IFU (45). However, no consensus exists among clinical and preclinical studies on the optimum kind of pericytes to be utilized nor is there any proven optimal method or technique of pericyte treatment for IFU. However, our findings based on our STZ-induced diabetic mouse model suggest that pericytes may slow the course of IFUs in diabetic patients by delaying HUVEC senescence and promoting wound healing *via* increased vascularization. Our results indicate that pericytes may enhance wound healing, consequently making pericyte transplantation suitable for treating IFUs.

## Data Availability Statement

The original contributions presented in the study are included in the article/supplementary material, further inquiries can be directed to the corresponding author.

## Ethics Statement

The animal study was reviewed and approved by CHA Administrative Panel on Laboratory Animal Care.

## Author Contributions

KK, H-JA, S-HK, JK, JL, SP, HL, and IJ were responsible for the data curation. KK, H-JA, JK, and S-HK were responsible for the formal analysis. KK and JK were responsible for writing the original draft. H-JA and CS were responsible for the manuscript review and editing. SL was responsible for the conceptualization, funding acquisition, investigation, methodology, manuscript review, and editing. All authors contributed to the article and approved the submitted version of the manuscript.

## Funding

This study was supported by a National Research Foundation (NRF) of Korea grant funded by the Korea government (MSIT) (Nos. 2021R1G1A109434111 and 2021R1A4A3023587) and by the Korean Fund for Regenerative Medicine funded by the Ministry of Science and ICT and Ministry of Health and Welfare (HH21C0013).

## Conflict of Interest

The authors declare that the research was conducted in the absence of any commercial or financial relationships that could be construed as a potential conflict of interest.

## Publisher's Note

All claims expressed in this article are solely those of the authors and do not necessarily represent those of their affiliated organizations, or those of the publisher, the editors and the reviewers. Any product that may be evaluated in this article, or claim that may be made by its manufacturer, is not guaranteed or endorsed by the publisher.
